# Childhood-Onset Psychosis: A large UK case series

**DOI:** 10.1007/s00787-025-02895-3

**Published:** 2025-10-27

**Authors:** Mitra S. Sato, Anthony James, Marinos Kyriakopoulos, Anna C. Need

**Affiliations:** 1 Faculty of Medicine, Department of Brain Sciences, Imperial College London, Du Cane Road, London, UK; 2 Department of Psychiatry, University of Oxford, Warneford Hospital, Oxford, UK; 3 1st Department of Psychiatry, National and Kapodistrian University of Athens, Eginition Hospital, Athens, Greece; 4 Department of Child and Adolescent Psychiatry, Institute of Psychiatry Psychology and Neuroscience, King’s College London, London, UK; 5 South London and Maudsley NHS Foundation Trust, London, UK

**Keywords:** Childhood onset psychosis (COP), Childhood onset schizophrenia (COS), Very early onset psychosis (VEOP), Ethnically diverse, Comorbid neurodevelopmental disorders, Treatment resistant

## Abstract

Childhood-Onset Psychosis (COP) is an extremely rare and severe variant of psychosis. This paper presents the largest UK case series of COP, comprising 39 families (105 individuals) recruited between 2016 and 2019. It is also the most ethnically diverse collection of COP cases worldwide, with 36% European ancestry, 23% South Asian, 18% African, 10% Mixed ancestry and 13% Other. All probands were hospitalised and diagnosed with a psychotic disorder by age 13, including schizoaffective disorder, paranoid schizophrenia, childhood-onset schizophrenia and other nonorganic psychotic disorders. The cohort had a male-to-female ratio of 1:1.4, with females diagnosed at a higher mean age (11 years 9 months) than males (10 years 6 months). While 54% of probands had no reported first-degree family history of mental disorders, 59% presented with comorbid neurodevelopmental conditions. Approximately one-third were treated with clozapine, reflecting the severity and treatment resistance of the cases.

**Supplementary Information:**

The online version contains supplementary material available at 10.1007/s00787-025-02895-3.

## Introduction

Childhood-onset psychosis (COP) broadly referring to the onset of psychotic symptoms at age 13 or younger, remains diagnostically and therapeutically challenging [1–5]. While various terms have been used in the literature – such as childhood-onset schizophrenia (COS) or very early-onset psychosis (VEOP) – we use COP as an umbrella term to refer to this clinical entity. Cases are diagnosed based on the adult schizophrenia criteria as outlined in DSM or ICD classifications [5–10]. COS incidence is generally accepted to be less than 0.04% [1]. UK surveillance data report an annual incidence of nonaffective psychoses in children aged 13 or younger at approximately 0.21 per 100,000 [11]. The early onset of illness is associated with distinct clinical features and a more complex prognostic course [1, 8, 10, 12–16].

The symptomatology of COP is notably severe, with auditory hallucinations being a prominent feature [17–25]. Visual and tactile hallucinations, delusions, thought disorder, and negative symptoms such as affective flattening and withdrawal are also significant [3, 12, 17, 19, 20, 24–26]. These symptoms alongside suicidal behaviours often lead to hospitalisation [18, 27].

Diagnosing COP is difficult [7, 27]. Hallucinations can be part of the normal developmental spectrum [7, 27, 28], yet they are also present in a range of conditions including autism [29] and affective disorders [30]. A key to diagnosis lies in the persistence and severity of symptoms and their impact on function [13, 31–33], a task complicated by the developmental variability in symptom expression across childhood and adolescence [34].

The rarity of COP is reflected in the lack of large, representative cohorts. One major exception is the landmark cohort established by the National Institute of Mental Health’s (NIMH), which remains the most comprehensive study of COS to date. From over 3000 referrals, 121 cases were rigorously confirmed through stringent outpatient screening, prolonged inpatient observation (including medication washout) and repeated diagnostic evaluations, ensuring a high degree of diagnostic precision and phenotypic clarity [6, 8, 27]. This pioneering work has significantly advanced our understanding of COS, providing invaluable clinical, neuroimaging and genetic insights that continue to inform the field. However, due to its strict inclusion criteria, the cohort may underrepresent the broader spectrum of COP, particularly individuals with co-occurring intellectual disability. A study from Nigeria [35] included 33 COP cases, while other case series report much smaller numbers [36–39]. A series of genomic findings from COS patients have been reported across different studies, including over 80 cases from the US [40–42], 37 from Israel [43] and 10 from China [44], however, no overview of clinical and demographic characteristics were provided.

Comorbid conditions are frequent in COP [5, 25, 45, 46], ranging from mood disorders [12] and ADHD [1] to autism spectrum disorder [46, 47] to profound language and motor developmental delays [1, 25]. Such comorbidities lead to phenotypic heterogeneity and are prognostically significant, with earlier and more severe premorbid disturbances correlating with worse outcomes [2]. Neurobiologically, COP is continuous with AOS [6–9], sharing similar structural brain abnormalities and cognitive deficits [2]. COP, however, has more pronounced cognitive and developmental disturbances [6–9].

The treatment of COP remains challenging, as antipsychotics – while foundational to symptom management – demonstrate only partial efficacy and carry significant risk profiles, particularly in children and adolescents [10, 48–52, 54–56]. Although second-generation antipsychotics (SGAs) are preferred for children and adolescents due to their more tolerable side effects [58] they continue to frequently exhibit greater susceptibility to metabolic dysregulation, weight gain, prolactin elevation, sedation, cardiovascular effects and extrapyramidal symptoms than adults [54–57, 59–61]. It remains unclear whether antipsychotics confer intrinsically lower benefit in youth [62, 63] although the generally more severe and treatment-resistant course associated with earlier onset may give the appearance of diminished response compared to adults. Notably, the evidence base for maintenance antipsychotic therapy beyond eight weeks in youth is scant, yet continued use is often justified in cases of poor adherence or severe illness course [64–66]. Clozapine has demonstrated superior efficacy in early-onset schizophrenia refractory to two adequate antipsychotic trials, with open-label follow-up confirming sustained symptom improvement, although its use mandates vigilant monitoring for agranulocytosis, seizures, myocarditis and gastrointestinal hypomotility [51, 62, 67, 68]. Given these pharmacological limitations, comprehensive treatment plans must integrate psychosocial interventions – such as cognitive remediation therapy, community-based rehabilitation and family support – which recent trials have shown to enhance cognitive and functional outcomes in adolescents with COP [39, 69, 70].

The heterogeneity of COP in the clinic, in contrast to the careful curated cohorts that are used for clinical research, combined with the likelihood that any individual clinician may only see one or a few COP patients in their career, means that the diagnosis and treatment of COP cannot easily be guided by published literature. This paper aims to help fill this gap by providing data from a cohort of patients diagnosed with COP in the UK.

## Methodology

### Study design

Between 2016 and 2019, families of children diagnosed with a psychotic disorder before the age of 13 were recruited via clinician referral from Tier 3 and Tier 4 Child and Adolescent Mental Health Services (CAMHS) across England. Feasibility assessments were conducted with 24 NHS Trusts to enable nationwide recruitment. Referrals were made by consultant child and adolescent psychiatrists following confirmed diagnoses of psychosis. Although hospitalisation was not an inclusion criterion all cases had required inpatient care due to illness severity. Referrals included both inpatient and outpatient cases and individuals were eligible regardless of the time since diagnosis. Written informed consent (and age-appropriate assent) was obtained. Diagnoses were verified through detailed review of clinical records; no research-administered diagnostic instruments were used. All cases had been assessed by multiple CAMHS psychiatrists as part of standard NHS care. Phenotypic data was collected via interviews. Additional family members (siblings, parents and other affected relatives) were included where possible. Ethical approval was granted (REC reference 15/SC/0206; IRAS ID 170402).

### Inclusion and exclusion criteria

All participants provided consent if over 16 and assent if under 16 with parental consent. The study obtained ethical approval to include adult relatives (including parents) with intellectual disabilities or other conditions affecting informed consent. Exclusion criteria included prematurity (birth weight < 1500 g), brain injury (e.g., perinatal hypoxia), substance abuse prior to diagnosis and known medical conditions or severe abuse that could explain the psychosis.

### Data collection

Data collected from consented participants included:

- *Medical Records*: Mental, neurological, and general health history. Diagnoses were made in accordance with the Multiaxial ICD-10 classification of child and adolescent mental disorders (World Health Organization, 1996) and had been established prior to study entry by treating NHS psychiatrists. The research team did not re-assess diagnoses but confirmed them through review of clinical records which included multidisciplinary evaluations, mental disorder history and in all cases at least one inpatient admission due to illness severity.
- *Cognitive Testing*: Post-diagnosis IQ data were obtained from descriptive entries in medical records or from direct Wechsler Abbreviated Scale of Intelligence II (WASI-II) assessments conducted as part of the study. When historical IQ results were available these were used directly; otherwise, WASI-II assessments were administered. Scores below 70 (WASI-II classification: “extremely low”) were categorised as indicating intellectual disability (ID).
- *Interviews*: Interviews were conducted to collect developmental history, family mental disorder history and relevant environmental exposures. When one parent was unavailable during initial contact, follow-up interviews were conducted.

#### Statistical analysis

All analyses were performed using R (version 4.2.2). Continuous variables were summarised as means +/- standard deviations (SD) and compared using the non-parametric Mann-Whitney U test. Categorical variables were reported as frequencies and percentages with group comparisons assessed using chi square tests. Missing data were excluded. Statistical significance was set at *p* < 0.05. Data visualisations were generated using ggplot2 and related R packages.

## Results

### Demographics

A total of 105 individuals were recruited including 39 probands with COP. Demographic characteristics are summarised in Table 1. Males were diagnosed at a significantly younger age (10.6 ± 1.9 years) than females (11.9 ± 1.5 years; *p* = 0.037). Onset type also differed significantly between sexes (*p* = 0.046) with females more likely to present with acute onset (61%) compared to males (44%). Ancestry and immigration status did not significantly differ between sexes (*p* = 0.256 and *p* = 0.613, respectively).

**Table 1:** **Demographics of the cohort**. Continuous variables are presented as mean ± standard deviation (SD) (Mann-Whitney U test), categorical as frequencies (%) (Chi-squared). “Mixed Afr and Eur” refers to one European and one African parent. “Other” includes Middle Eastern, East Asian and Hispanic. Significant results marked *.

Characteristic	Males (*n* = 16, 41%)	Females (*n* = 23, 59%)	Total (*n* = 39)	*p*-value (test)
Mean age at diagnosis (years)	10.6 ± 1.9	11.9 ± 1.5	11.3 ± 1.8	0.037* (Mann-Whitney U)
Onset type (%)				0.046* (chi squared)
Acute	7 (44)	14 (61)	21 (54)	
Insidious	9 (56)	9 (39)	18 (46)	
Ancestry (%)				0.256 (chi squared)
European	5 (31)	9 (39)	14 (36)	
South Asian	2 (12)	7 (30)	9 (23)	
African	5 (31)	2 (9)	7 (18)	
Mixed Afr and Eur	1 (6)	3 (13)	4 (10)	
Other	3 (19)	2 (9)	5 (13)	
Immigration				0.613 (chi squared)
First generation	2(12)	1(4)	3(8)	
Second generation	9(56)	13(57)	22(56)	
Non-immigrant	5(31)	9(39)	14(36)	

**Fig. 1:**
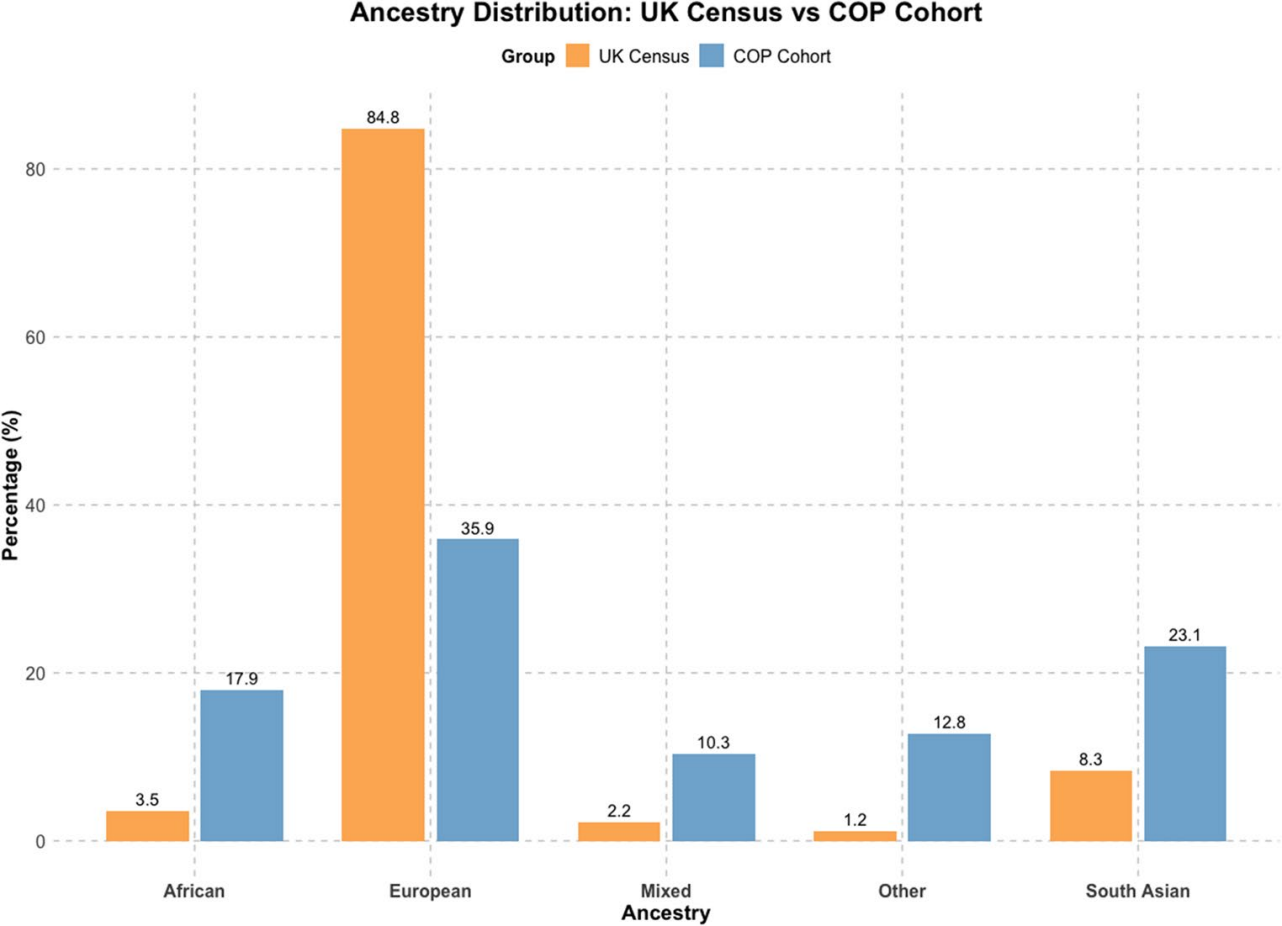
**Ancestry distribution**: Ethnic minorities are overrepresented, Europeans underrepresented compared to the UK census. Percentages above bars.

The cohort’s ancestry composition deviates from the UK population (Fig. 1).

### Diagnoses

All probands had a primary psychotic disorder diagnosis and were hospitalised at least once. The split of diagnoses, along with the prevalence of positive and negative symptoms is detailed in Table 2.

**Table 2:** **Diagnoses and symptom frequencies**. Diagnoses and symptoms are presented as frequency (n) and proportion (%) of the total count (*n* = 39).

Category	Sub-category	Frequency (*n*)	Proportion (%)
Diagnoses			
	Other nonorganic psychotic disorders	21	54
	Schizoaffective disorder	8	21
	Schizophrenia	6	15
	Bipolar disorder with psychotic symptoms	2	5
	Delusional disorder	1	3
	Depression with psychotic symptoms	1	3
Positive symptoms			
	Hallucinations (all types)	39	100
	Auditory	37	95
	Visual	31	79
	Olfactory	4	10
	Somatic	3	8
	Tactile	1	3
	Delusions (all types)	32	82
	Capgras	6	15
	Persecutory	5	13
	Grandiose	4	10
General symptoms			
	Behavioural difficulties	36	92
	Anxiety	36	92
	Sleep disturbances	29	74
	Suicidal ideation	10	26

### Family history

Most cases were sporadic, with no first-degree relative diagnosed with a mental illness (22/39, 56%). Those with at least one parent diagnosed with schizophrenia accounted for 8/39 (21%) while the remaining 9/39 (23%) had a parent with a mental disorder other than schizophrenia, including depression (*n* = 7) and anxiety disorder (*n* = 2). Family history was further analysed (Suppl. Figure 1). There was no significant difference when stratified by diagnostic age group (χ^2^ = 0.09, *p* = 0.95) or by sex (χ^2^ = 0.01, *p* = 0.99).

### Intellectual functioning

**Fig. 2:**
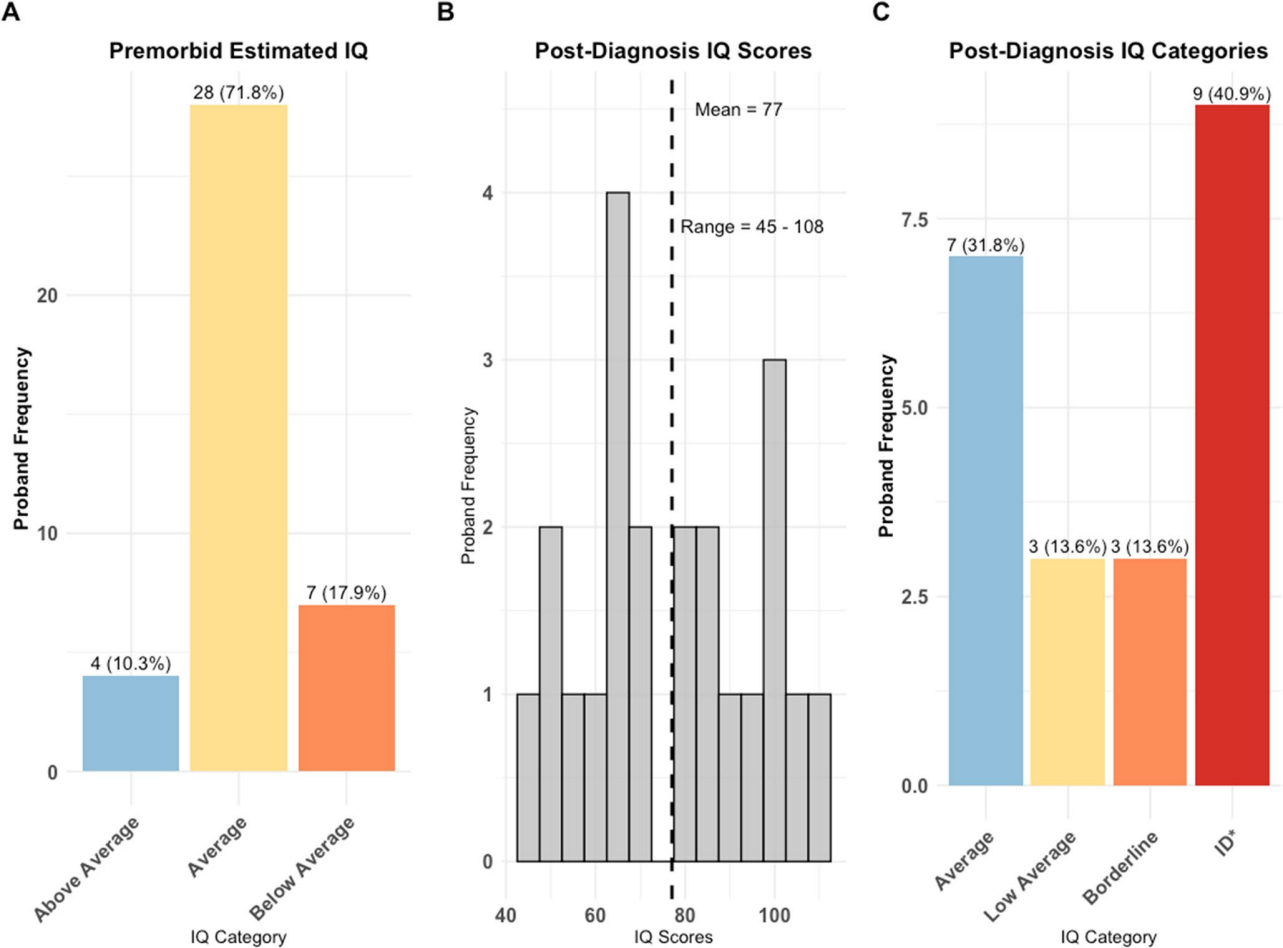
**Premorbid and post-diagnosis IQ distribution**. (**A**) distribution of premorbid estimated IQ categories based on school records. (**B**) distribution of post-diagnosis IQ scores (*n* = 24). (C) post-diagnosis IQ categories. *ID = see data collection section

Premorbid IQ was unavailable for most cases therefore school reports and parental interviews were used to categorise them as having average, above average or below average cognitive functioning (Fig. 2A). Post-diagnosis IQ scores were available for 24 probands, assessed using prior medical records or WASI-II at recruitment (Fig. 2B-C). IQ data for 15 probands were unavailable due to missing records or testing constraints. Post-diagnosis IQ was significantly lower in individuals with premorbid developmental delay (t-test, *p* = 0.033, suppl. Figure 2 A) but not significantly impacted by comorbidities (suppl. Figure 2B). Speech and language delay were present in 46% (*n* = 18) of probands.

### Antipsychotic treatment

At the time of recruitment, all probands were taking antipsychotics, with 5 individuals (12.8%) receiving two concurrently. Clozapine was most frequently prescribed (*n* = 14, 35.9%), and appeared more frequent in probands diagnosed at an older age (Suppl. Table 1); however, this was not statistically significant (χ²=1.18, *p* = 0.55). Olanzapine was prescribed in 9 cases (23.1%), followed by risperidone (6 probands, 15.4%), and aripiprazole and quetiapine, each in 4 probands (10.3%). Mood stabilisers were recorded in 6 individuals (15.4%), including sodium valproate, lithium carbonate, and carbamazepine. Antidepressants were prescribed in 7 cases (17.9%), with sertraline and fluoxetine most frequently represented. Sleep aids, primarily melatonin, were used in 6 probands (15.4%). Adjunctive therapies were common, including antidiabetic medications (5 cases, 12.8%), laxatives (3 cases, 7.7%), anticholinergics (3 cases, 7.7%), and vitamin supplements. These findings highlight the pharmacological complexity often required in this population.

### Age and diagnostic delay

**Fig. 3:**
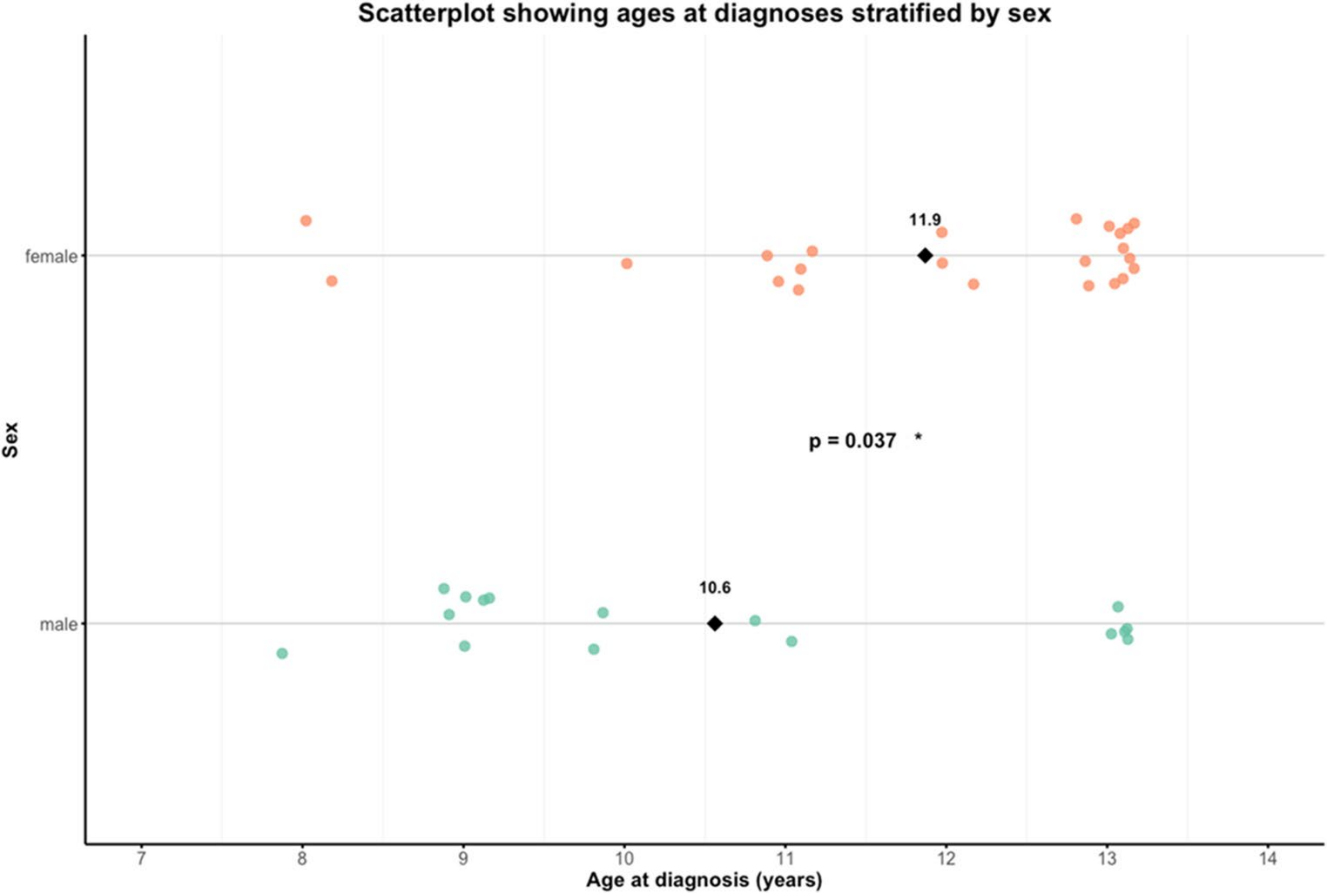
**Age at diagnosis by sex**. Individual ages at diagnosis, stratified by sex. Black diamonds indicate mean ages (male: 10.6 years; female: 11.9 years). The difference is statistically significant (*p* = 0.037, Mann-Whitney U-test).

**Fig. 4:**
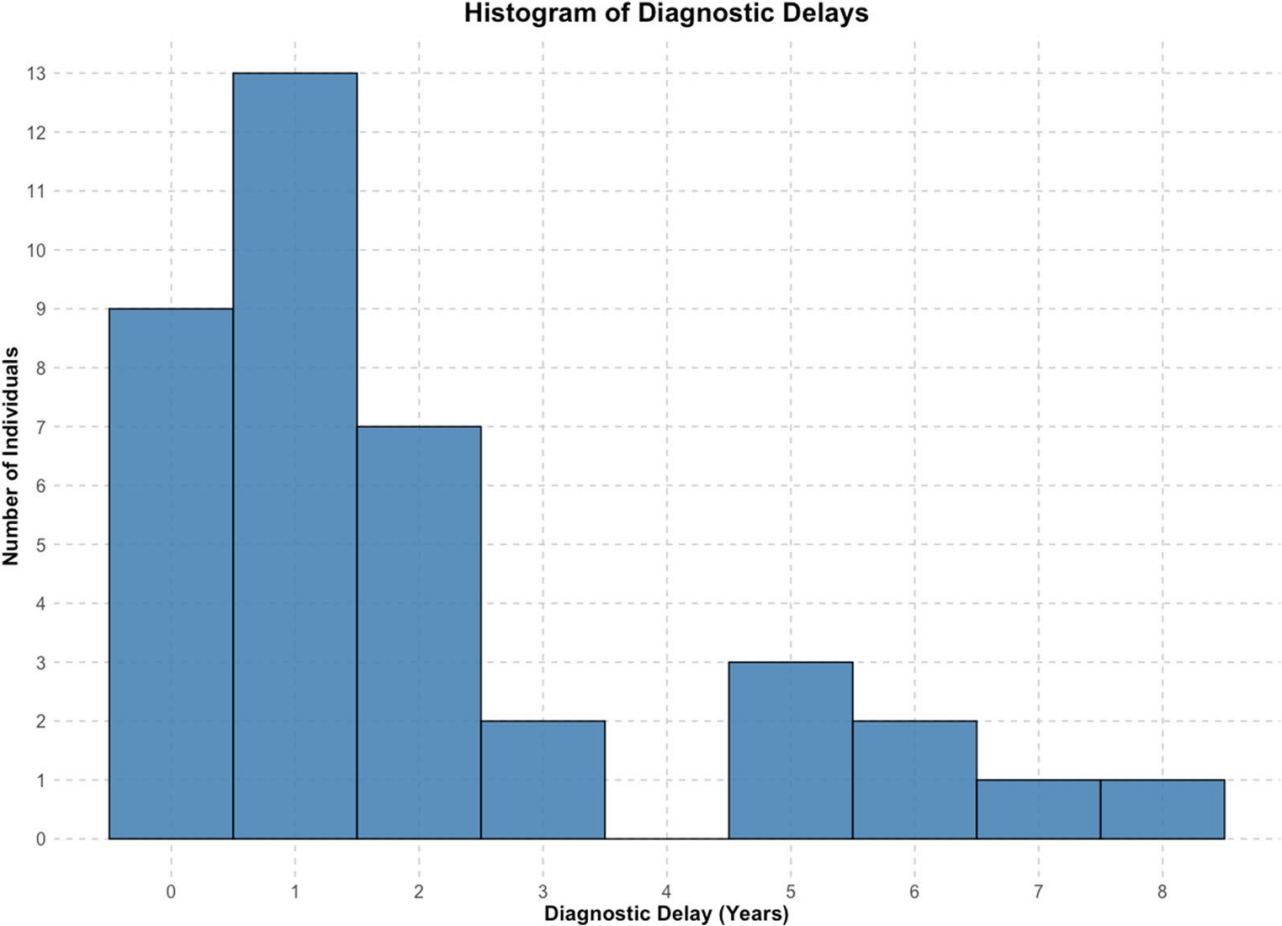
**Distribution of diagnostic delays**. The number of individuals by diagnostic delays in years.

Males were diagnosed significantly earlier than females (*p* = 0.037, Mann-Whitney U; Fig. 3), though symptom onset ages showed no sex differences (mean: 9.4 years; *p* = 0.63). This suggests possible diagnostic delays may underlie sex differences in diagnostic age. The majority of the cohort were diagnosed within two years of symptom onset (*n* = 23, 59%) though delays of up to eight years occurred (Fig. 4). Prolonged delays (≥ 5 years, *n* = 7) were more common among females.

## Discussion

This study presents a large case series of childhood onset psychosis collected in England between 2016 and 2019. Compared with published incidence estimates from the only other UK study [11], which identified 8 confirmed cases per year, our cohort of 39 cases over 3 years (~ 13 cases per year) suggests a high level of case ascertainment and likely captures the majority of new incidences. Although not all patients were newly diagnosed at recruitment, the scale and breadth of identification – through national inpatient services and outpatient referrals – makes this one of the largest datasets assembled for this rare condition nationally. Unlike other studies [1, 8, 27], this dataset prioritises inclusivity and reflects childhood psychosis as seen in community and clinical settings. While the size of the cohort highlights the scale of recruitment effort, it remains likely that some cases were not identified due to underdiagnosis, challenges in clinical recognition, delayed referrals, or barriers to research participation, as discussed below. Despite these limitations, this cohort provides valuable insight into better understanding of COP within the UK healthcare and population setting.

The cohort’s ethnic makeup differs from the general UK population [71], with White British underrepresented (Fig. 1) and a notable presence of minority European groups. Studies show that white ethnic minorities face a 50% increased risk of psychosis compared to White British individuals likely due to migration stress and socioeconomic challenges [72, 73]. Notably, nearly a third of the cohort is of South Asian ancestry, consistent with a 2–4 times higher risk of psychosis reported in this group [72]. Almost half the cohort are reportedly first- or second-generation immigrant families. Among first-generation immigrant families, early childhood migration (before age 2) was observed in 2/3, a critical period linked to increased psychosis risk [74–77]. Second-generation immigrants, comprising over a third of the cohort, showed a higher prevalence of psychosis, aligning with studies indicating a twofold increased risk compared to non-immigrants [78, 79]. Although consistent with known psychosis risk factors, comparisons with the general population should be made cautiously, given the non-random sampling and potential biases related to diagnosis, healthcare and research access. Nonetheless, the alignment with existing epidemiological evidence highlights the relevance of ancestry and migration-related exposures in COP.

Family history was broadly similar across the cohort (Suppl. Figure 1) with 44% (17/39) reporting a parent with schizophrenia or another mental illness – higher than the 33% previously reported for COS [80], likely due to broader inclusion criteria in this study. Most cases (56%) were sporadic or involved extended family neurodevelopmental or mental disorder histories, suggesting a mix of gene and socioenvironmental contributors [81]. Twin and family studies estimate schizophrenia risk in first-degree relatives of COS cases at ~ 10%, compared to ~ 1% in the general population [13]. Genetic studies support a higher burden of risk variants in COS: pathogenic CNVs are found in 11.9% of cases vs. 2.5% in AOS [82], and many overlap with those found in neurodevelopmental disorders such as ASD, ID, DD and epilepsy [83]. Sequencing studies reveal enrichment of *de novo* and rare inherited variants in genes associated with neurodevelopment [40, 43, 84–91], supporting a genotype-first model where mental disorder and neurodevelopmental conditions reflect a variable expression of shared genetic risk [92]. In line with this, exome sequencing in COS families has identified likely pathogenic variants in 19% (7/37) of cases, highlighting the potential value of incorporating genetic testing into early diagnostic pathways [84].

Consistent with this neurodevelopmental model, cognitive impairment was a prominent feature in the cohort with indications that participants might perform more poorly on IQ assessments following diagnosis. Males showed greater decline than females (Fig. 2) with larger drops in those with developmental delays (Suppl. Figure 2 A), consistent with the literature [93–95]. Poorer premorbid functioning, linked to worse prognoses [96–99] may explain these findings. This pattern aligns with reductions in global cognitive functioning seen in COS [100–102], attributed by the NIMH group to disrupted brain connectivi, particularly in social-cognitive and sensorimotor regions [103]. However, interpretation of the IQ scores is constrained by methodological limitations. Given the scores were derived from medical records or home-based assessments, reliability could potentially be impacted [104]. Additionally, the lack of a systematic socio-economic status evaluation in this cohort, a known influence on IQ measurement [105], further restricts conclusions.

Clozapine was prescribed to 38.5% (*n* = 15) of the cohort following inadequate responses to at least two antipsychotics, consistent with its role as a third-line treatment [106–108]. Females were twice as likely as males to receive clozapine, particularly those with diagnostic delays potentially due to their older age at diagnosis. Adult studies show lower clozapine rates in females [109], while adolescent studies report no gender disparities [110]. Clozapine’s use in youth is approached cautiously due to side effects with 40% of UK psychiatrists avoiding it in under 18 s [111] (Suppl. Table 1). Polypharmacy was common, with antidepressants (e.g., sertraline) and mood stabilisers (e.g., sodium valproate) prescribed to manage comorbid symptoms, possibly aligning with practices aimed at improving symptom management [112, 113]. Despite limited data in the younger populations, there are reports of increased risk of adverse drug reactions with psychotropic polypharmacy [114]. These findings reflect the complexity of pharmacological management in COP and underscore the need for integrated treatment approaches. While clozapine remains a key option for treatment-resistant cases [1, 27, 52, 53], its use should be complemented by psychological interventions such as cognitive-behavioural therapy (CBT), family therapy, and educational support [6].

Diagnostic patterns highlight gender differences, with females more often diagnosed at age 13, while diagnoses before this age were evenly distributed. In younger probands, males frequently presented with externalising behaviours, such as ADHD or learning disabilities. The literature [115–121] suggests that such behaviours in males are more likely to prompt earlier clinical attention, whereas internalising symptoms more typical of females, such as ASD traits, anxiety and OCD as seen in this cohort, may be misattributed to stress, trauma or affective disorders. This may contribute to delayed recognition of psychosis in girls, particularly in the absence of overt behavioural disturbance.

Capgras delusions were identified in 6 of 39 probands (15%), a notably high rate given that only 38 paediatric cases have been reported globally in the past decade [122]. This likely reflects both historical under-recognition and limited investigation in paediatric populations. A recent report describes Capgras in the context of complex presentations, such as comorbid obsessive-compulsive symptoms and emotional dysregulation [123]. In this cohort, targeted exploration of delusional subtypes through clinical notes and parental interviews may have enhanced detection, suggesting the possibility that these types of delusions could be more prevalent in COP than previously thought.

Stigma around mental illness, particularly in minority communities [124], can delay diagnosis as families may avoid seeking help or attribute symptoms to non-medical causes. In two cases, care was initially sought abroad due to cultural or religious beliefs, delaying local treatment. South Asian ancestry was similarly represented across age groups with females consistently outnumbering males. Cultural stigma, especially in South Asian families may explain the older diagnostic age for some probands aligning with reports of mental health stigma in this community [125]. More broadly, cultural norms often socialise females to mask emotions whilst males express visible behaviours [126, 127], potentially contributing to later diagnoses in the females in this study.

This study’s greatest strength lies in its inclusivity, distinguishing it from others that often exclude individuals with intellectual disabilities or non-English speakers [8]. By capturing the majority of families with affected children we ensured representation of groups frequently left out of research. Ethical approval allowed personal consultees to provide consent for participants with intellectual disabilities. Language barriers were addressed through interpreters and translated materials, and home visits were conducted nationwide for families unable to travel to reduce burden. Instead of lengthy diagnostic tools like K-SADs [128], targeted questions and use of medical records ensured efficient data collection, with follow-ups conducted when one parent was unavailable. These efforts produced a robust, diverse dataset – setting a new standard for inclusivity in research.

However, there are several limitations. First, inconsistencies in IQ testing conducted at varying ages and under different conditions make it difficult to distinguish true cognitive decline from age-related differences in test performance. Second, while our inclusive recruitment approach is a strength, it may have impacted data quality. Efforts to address language barriers using interpreters and translated materials, while effective, risked losing subtle cultural nuances. Despite tailored questionnaires, this may have affected data granularity. Third, cultural factors, including varying parental beliefs about mental disorder symptoms, likely influenced symptom reporting and potentially reporting of relevant family history, introducing sociocultural variability into the dataset. Fourth, the absence of standardised diagnostic interviews such as K-SADs may limit comparability with other studies. However, diagnostic stability was ensured as all had been CAMHS inpatients during their clinical care, had undergone multiple assessments by experienced clinicians and had diagnoses confirmed using operationalised diagnostic criteria. Fifth, excluding participants with significant prematurity or birth hypoxia may have inadvertently excluded those with undiagnosed genetic disorders causing both birth complications and psychosis. Finally, this was an exploratory study without a priori statistical analysis plan. Multiple post hoc comparisons were conducted to identify potential trends and, as such, findings, particularly sex differences, should be interpreted with caution given the risk of type I error and the limited statistical power inherent to the small sample size.

In conclusion, this study presents the largest and most ethnically diverse cohort of COP cases of unknown cause in the UK. By inclusively recruiting and excluding only cases with clear non-genetic causes, we likely captured the majority of affected families. Despite its diversity, the cohort’s clinical presentation aligns closely with global COP studies, reflecting consistent core clinical features across socially diverse populations.

## Supplementary Information

Below is the link to the electronic supplementary material.

Supplementary Material 1 (DOCX. 294 KB)

## Data Availability

The analyses were conducted using detailed clinical data that are not publicly available due to patient confidentiality. No additional data are available.
