# Supplementary material for: Childhood-Onset Psychosis: A large UK case series
Source: Eur Child Adolesc Psychiatry. 2025 Oct 27;35(3):899–910. doi: 10.1007/s00787-025-02895-3 (PMC13212701; doi:10.1007/s00787-025-02895-3)
Supplement: Supplementary file 1 — Supplementary Material 1 (DOCX. 294 KB) [file 787_2025_2895_MOESM1_ESM.docx]

#### Supplementary tables and figures for the cohort paper


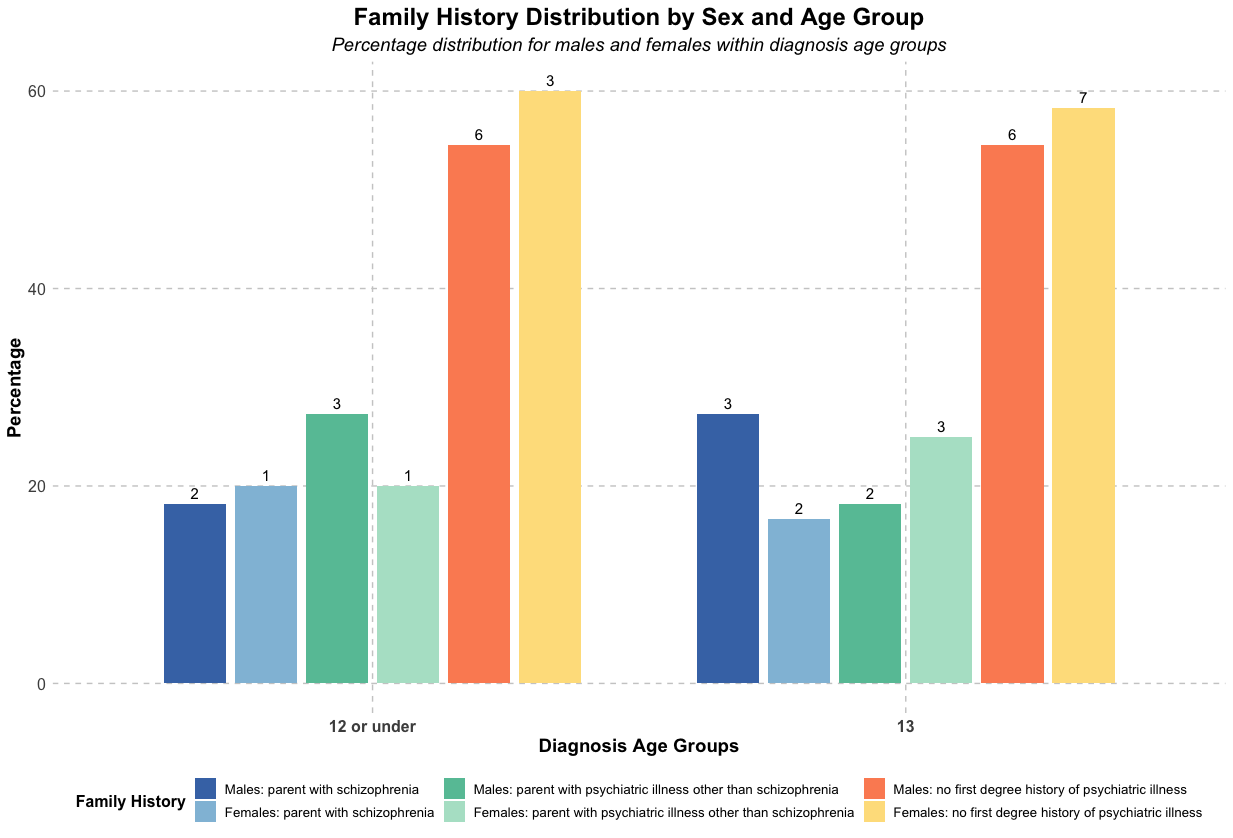


Supplementary Figure 1. Distribution of family history by diagnostic age group and sex. Bar chart showing the percentage distribution of first-degree family history. Numbers above bars indicate actual counts.


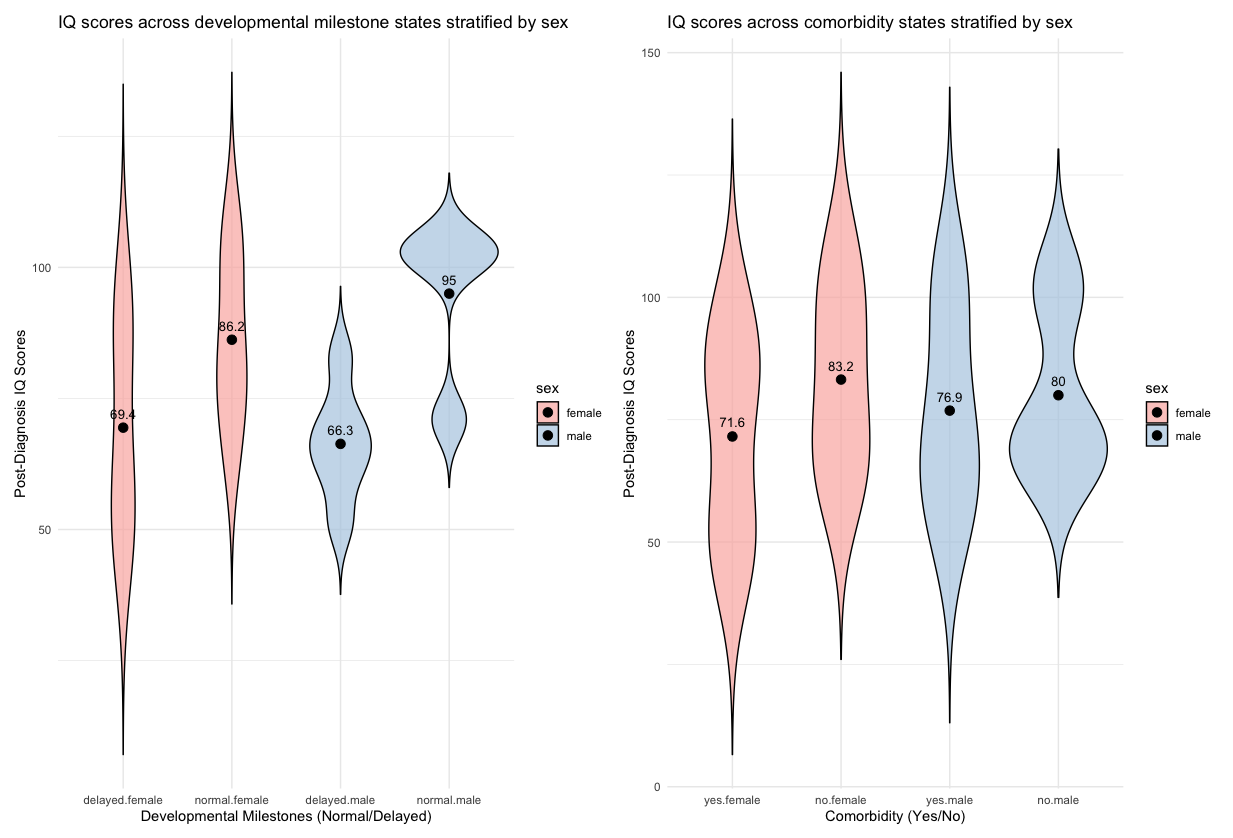
Supplementary Figure 2. Post-diagnosis IQ scores stratified by (A) developmental milestone status and (B) comorbidity status, split by sex.

Developmental milestone status was derived from a combination of clinical records and parental interviews conducted at the time of recruitment. Classification of ‘delayed’ milestones was based on either a recorded symptom e.g. speech delay/ developmental delay or parental reports of early speech, motor or other developmental concerns.

This plot suggests that individuals, particularly males with a history of delayed developmental milestones showed lower post-diagnosis IQ scores compared to those without delays. A similar pattern was observed for individuals with comorbid diagnoses though the magnitude of the difference between comorbid and non-comorbid groups appeared smaller.


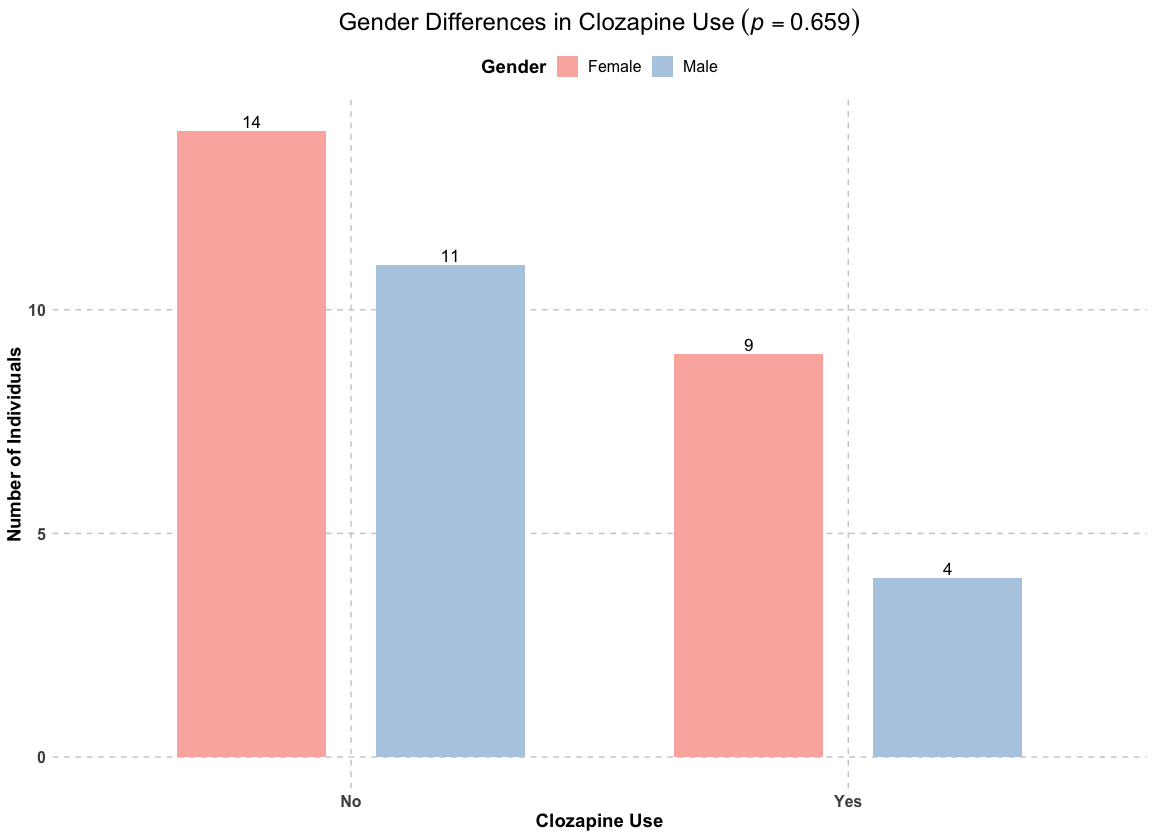
Supplementary Figure 3. Clozapine use by sex. The y-axis shows the numbers of probands with observed counts displayed on the bars. Statistical comparison yielded nonsignificant differences (X^2^=0.195, p=0.659) in clozapine use between genders.

| Age at Diagnosis | On Clozapine (M/F) | Not on Clozapine (M/F) | % On (of age group) | % Not on (of age group) |
| --- | --- | --- | --- | --- |
| 8-9 | 2/0 | 5/2 | 22% | 78% |
| 10-11 | 0/3 | 4/3 | 30% | 70% |
| 12-13 | 2/6 | 2/9 | 42% | 58% |

Supplementary Table 1. Counts and percentages of individuals stratified by age at diagnosis, clozapine treatment status (on vs not on) and sex. Each cell shows the number of males/females in each group. Percentages reflect the proportion of individuals within each age group who were on or not on clozapine. group.

| Age at Diagnosis | With Comorbidity (M/F) | Without Comorbidity (M/F) | % With (of age group) | % Without (of age group) |
| --- | --- | --- | --- | --- |
| 8-9 | 5/2 | 2/0 | 78% | 22% |
| 10-11 | 3/3 | 1/3 | 60% | 40% |
| 12-13 | 3/10 | 2/5 | 65% | 35% |

Supplementary Table 2. Counts and percentages of individuals stratified by age at diagnosis, comorbidity status (with vs. without), and sex. Each cell presents the number of males/females in each category. Percentages reflect the proportion of individuals within each age group who had or did not have comorbidities. A chi-square test (χ² = 0.0, p = 1.0) showed no statistically significant difference in the distribution of comorbidities between males and females, suggesting an even distribution of comorbidities across sexes within this cohort.
